# The Etiology and Management of Dental Implant Failure: A Review

**DOI:** 10.7759/cureus.30455

**Published:** 2022-10-19

**Authors:** Shraddha P Kochar, Amit Reche, Priyanka Paul

**Affiliations:** 1 Public Health Dentistry, Sharad Pawar Dental College, Datta Meghe Institute of Medical Sciences, Wardha, IND

**Keywords:** re-implantation, failed implant, implant survival, implant restorations, endosseous dental implants

## Abstract

As the world is embracing technology, dental technology is merging with artificial intelligence. Dentists are striving to perfect the art of placing dental implants. Implants for the rehabilitation and retention of dental and facial prostheses have graduated from a phase of wishful thinking to one of the most gratifying experiences for patients and treating fraternity alike. Implants and restorations supported by implants have a good long-term survival percentage. Complications and implant failure, which can still happen, are seen by many clinicians as significant barriers to implant treatment. Implant therapy still involves a biological healing and integration process despite recent advancements. These biological processes are complex and may be hampered by local or systemic factors, which could result in problems and implant failure. For the implant surgeon and dental professional, it is crucial to manage patients who have certain risk factors and be able to address potential complications and failure. The aim of this article is to discuss frequent complications of implant failure and its management and help clinicians in placing and restoring implants less painfully and vicariously receive some valuable experience.

## Introduction and background

Dental implant growth throughout history is a rich and fascinating time travel adventure. Dental implants have been used by people to repair missing teeth in one way or another since the dawn of civilization. Dr. Per-Ingvar Brånemark unintentionally discovered that titanium implants had a higher success rate in 1952, which created the groundwork for current dental implants. He implanted a piece of titanium into the femur of a rabbit, but when he tried to remove it, the titanium had already fused with the bone [1]. After more testing, he successfully used titanium to restore a lost tooth for one of his patients. This was a significant development for the dental implant market. He then published a number of studies outlining the advantages of titanium use in dental implants. Dental implants have evolved over time to become what they are now thanks to Dr. Per-Ingvar Brånemark [2].

Endosseous dental implants have drastically changed how the teeth of individuals who are edentulous or partially edentulous can be restored. The high survival rates reported for single and multiple missing tooth replacements have demonstrated the effectiveness of implant-supported restorations as an approach for oral rehabilitation [3]. Implants can restore a single missing tooth without the need to replace the teeth next to it. Additionally, implants make it possible to provide fixed restorations for those who are completely or partially edentulous. Patients who have certain implants may experience failure within six months, severe bone loss, and problems and deformities that are irreversible [4].

From 4.3% after five years to 26.4% after 10 years, the frequency of connection-related problems (screw loosening or fracture) increased. Of the 9% of restorations that were cemented, loss of retention of the restorations occurred in 6.2% within five years and 24.9% within 10 years. Ironically, the “prevention” of these problems from happening is the “therapy” for the issue of an increasing incidence of complications. Complication risk can be decreased through improved case selection, awareness of systemic issues that may lead to complications, and better treatment planning [5]. The clinician can achieve more predictable planning, placement, and restoration of implant-supported restorations using available technology and diagnostic tools, such as computed axial tomography (CAT) scans, cone-beam (CB) scans, surgical guides, computer treatment planning, and aids to assess primary implant stability (i.e., Periotest and Osstell) [6].

## Review

The etiology of dental implant failure

Implant failure is most likely the result of multiple factors. Age and sex, smoking, systemic diseases, maxillary implant site, quantity and quality of bone, and implant surface treatments and features are some of the statistically examined parameters linked to implant failure. Dental implant failure is classified as early and late implant failure. Early implant failure means an implant showing clinical mobility before the placement of a final prosthesis. This is usually because of biological problems where the body does not accept the implant. It is called “rejection” of the dental implant. Early implant failure may be linked to immunological, genetic, and immunological variables. Late implant failure occurs within 1-3 years after the placement of the implant. Various factors associated with early and late implant failure are given in Table 1 [7].

**Table 1:** Causes of early and late dental implant failure Source: [7] AIDS: acquired immunodeficiency syndrome

Causes of early failure	Causes of late failure
Poor bone quality and quantity, systemic diseases such as uncontrolled diabetes mellitus, AIDS, osteoporosis, medications such as corticosteroids and bisphosphonates, smoking, infection, lack of primary stability, surgical trauma	Excessive loading, peri-implantitis, bruxism, teeth grinding at night time, retained subgingival dental cement, inadequate prosthetic construction, traumatic occlusion

Dental implant failure is also classified on the basis of local and systemic factors as well.

Local causes

The most frequent and avoidable cause of dental implant failure is infection. At any moment over the course of implant therapy, a bacterial infection that results in implant failures can happen. Peri-implantitis is a term used to describe an inflammatory response with bone loss in the soft tissues surrounding implants. The concept of peri-implantitis could include plaque-induced infection caused by plaque building up on the exposed surfaces of the biomaterial [8]. Although bacterial insult is the most prevalent cause of peri-mucositis, stress factors caused by a poor biomechanical environment are considered to be the cause of peri-implantitis. Fistulations, mucosal abscesses, and hyperplastic mucositis are other soft tissue problems that appear to be mostly infectious in origin [9]. In connection with loose prosthetic components, fistulas and hyperplastic mucositis are frequently observed. Food particles stuck in the peri-implant crevice can occasionally cause abscesses [10].

Implant failures associated with delayed healing are thought to be greatly influenced by the severity of the surgical trauma (lack of irrigation and overheating), micromotion, and several local and systemic features of the host [11]. Overload-related implant failures occur when the functional load placed on the implants is greater than what the bone can bear [12]. In addition to the severe loading circumstances, poor surgical technique, low bone quality, and poor prosthesis design are additional factors contributing to implant failures.

Management of Local Causes of Implant Failure

The initial step in treatment is to detect and diagnose the malfunctioning implant. Mobility, edema, discomfort, pus, bleeding, and radiographic evidence of peri-implant bone loss are the possible clinical indications and symptoms of implant failure. Any time there is obvious mobility following implant failure, the implant needs to be removed right away. To prevent further alveolar bone loss that would make the alternative of replacing the failed implant with a new one more challenging, it is crucial to recognize a failing implant as soon as possible. The patient should be motivated to perform an adequate level of plaque control on a regular basis. If an implant does fail or is unable to form bone around it, the most important thing is its rapid removal to avoid more bone loss because if the implant is left, more and more bone will be lost in order to place another implant. The possible treatment options for implant failure include the replacement of a faulty implant right away with one with a larger diameter, simultaneous replacement of failed implant with a guided bone regeneration (GBR) procedure, and a staged approach where the lost tissue is first rebuilt and the implant is then placed following site healing (delayed approach).

Peri-Implantitis

Peri-implantitis is a plaque-associated pathological disease that occurs in the tissues surrounding dental implants characterized by inflammation in the peri-implant mucosa and consequent gradual bone loss. Bacteria and food particles that build around dental implants and gum lines cause peri-implantitis. As a result, peri-implantitis often goes undiagnosed in its early stages. It is an inflammatory lesion that causes obvious bone loss; the marginal soft tissue will appear inflamed. Bacterial colonization is one of the prime factors that may be induced due to poor oral hygiene, cements retained in the subgingival area, and microscopic gaps between implant components [13]. Some of the signs of this condition include pain around the dental implant area, swollen lymph nodes, an unpleasant aftertaste, bleeding at the gum line, and slight movement of the dental implant [14].

Nonsurgical management of peri-implantitis: The first step in the nonsurgical management of peri-implantitis is mechanical cleaning of the implant surface with curettes of titanium or steel or prophy jets. Local medicaments that should be given include chlorhexidine chips (PerioChips), chlorhexidine lavage/citric acid or powder jet devices, local doxycycline or metronidazole gel, and Ligosan 260 mg (slow-release Ligosan or 12 days direct delivery) [15]. Laser decontamination can be done with either CO_2_ or erbium-yttrium-aluminum-garnet (YAG) laser of 1.5 w frequency. Photodynamic therapy, the photochemical decontamination of peri-implant tissues and the implant surface with a photosensitizer dye in combination with laser light, can be useful [16]. The treatment plan can include ozone therapy, which has proved useful in many studies. Systemic antibiotics that can be helpful are metronidazole 400 mg three times a day (TDS) + amoxicillin 500 mg TDS for seven days or clindamycin 300/600 mg four times a day for seven days [17].

Surgical management of peri-implantitis: Implants positioned in unsightly locations are typically only subject to surgical resection. The damaged implant is thoroughly debrided and decontaminated with the use of a surgical flap [18]. Surgery is done using membrane-covered autogenous bone grafts, autogenous bone grafts alone, membranes alone, and a control access flap procedure. The results showed that defects treated with membrane-covered autogenous bone grafts had significantly more bone regeneration and re-osseointegration than defects treated with the other three procedures [19].

Points to remember: There are some points that should be remembered while dealing with peri-implantitis cases. Patients should be thoroughly trained on proper oral hygiene techniques, paying particular attention to cleaning implant locations. Depending on the patient’s history and susceptibility to periodontitis, maintenance care should be given at least once a year [20]. Be aware that people who have had periodontitis are more likely to get peri-implant illnesses. Take preventative action if mucositis (bleeding) is seen near the implant [21]. Lesions should be treated as an ecological niche where anaerobic bacteria are present by probing them at a depth of 6 mm. Whenever the probing depth surrounding an implant is 6 mm or more, a radiograph should be obtained [22].

Management of Implant Fractures

An osseointegrated implant fracture is a catastrophic event that requires the removal of the remaining implant components. Infection from lingering germs, poisons, or pollutants that are present in the internally threaded area of the retained cracked implant may result from failure to accomplish this [23]. Rearranging the implant’s remnant to allow tissues to cover it or, if the implant was broken at a suitably low level, to allow tissues to repair over it may be prudent if the implant is adjacent to a critical structure, such as a neurovascular bundle or sinus cavity. One or more fractured implants may be recontoured, albeit very difficultly, and permitted to stay submerged if there are enough implants left to support the prosthesis [24]. This is known as a sleeping implant. This prevents surgery and added expense, time, and discomfort of implant removal. However, because of the risks associated with leaving a fractured implant in the bone, the patient’s informed consent and regular follow-up examinations to look for any new problems are required. If the implant is required for the support of the remaining prosthesis and no other site can be used, a skilled surgeon should carefully remove it, followed by grafting and/or re-entry at a later time.

Fractured implants can be removed by means of trephines. After this, a new implant can be placed at the same time. The dental surgeon should pay attention to the diameter of the trephine because it can affect the primary stability of the new implant. Apicoectomy is a useful procedure for simultaneously removing fractured implants and placing fresh implants. This method involved making a hole in the bone to better see the shattered implant’s apical fragments and remove those fragments via the hole. After that, a new implant is placed as usual, and the hole is filled with the patient’s own bone that was previously taken [25].

Points to remember: There are some points that are important to remember while dealing with cases of fractured implants. The length and diameter of the intended implants need to be taken into account when arranging therapy for a patient with incomplete dentition. To construct the prosthesis appropriately and prevent fracture, risk reduction may necessitate more implants. All patients who display parafunctional tendencies should wear occlusal guards. If possible, cantilevers or other unsupported prosthetic extensions should be avoided in the molar areas. Keep an eye out for severe bone loss and recurrent screw-loosening incidents.

Esthetic Complications and Management Due to Implant Malpositions

To get the best esthetic and functional results with implant therapy, proper 3D placement of implants is essential. One of the main causes of esthetic difficulties in implant dentistry is implant malposition in the esthetic zone. The three possible directions for implant malposition are mesiodistal, corono-apical, and oro-facial [26]. A malposition frequently consists of a variety of faults made in different directions. To avoid these issues, one must be aware of the aspects that the clinician and the patient both contribute to [27]. Implant malpositions can be corrected using the orthodontic bone stretching (OBS) technique, which involves deep partial osteotomies combined with heavy orthodontic forces. The applied force helps with esthetic rehabilitation by moving the implant axis and gingival line alignment toward the occlusal plane [28].

Points to remember: There are some points that are important to remember while treating esthetic complications due to implant malpositions. Ensure that the patient is aware of the dangers and esthetic implications of the procedure. Ideal esthetic results are frequently impossible to achieve because of pre-existing hard and soft tissue defects. The site’s hard and soft tissues should be carefully measured with respect to the intended implant position. The facial bone’s thickness should remain at 2 mm [29]. Ensure that the implant is positioned in the proper 3D location as defined by the restoration. The implant should be positioned in the apico-coronal plane (between 2 and 3 mm apical to the predicted mucosal boundary of the implant restoration), mesiodistal plane (at least 1.5 mm away from the roots of adjacent teeth), and orofacial plane (at the level of the gingival edge and 1.5 mm orally to the facial curve of the arch). If it is anticipated that it would be challenging to position the implant appropriately, a surgical guide stent should be taken into consideration. In cases where there are numerous lost teeth, surgical stents are strongly advised [30].

Complications and Management in Guided Bone Regeneration

The use of endosseous implants has been expanded to jaw bone regions with insufficient bone volume thanks to the advent of guided bone regeneration (GBR) in recent decades. The most frequent GBR consequence is seen as membrane exposure to the oral environment [31]. Wound dehiscence and membrane or mesh exposure can have a variety of effects, from a minor issue requiring membrane removal and resulting in incomplete bone growth to a major issue involving treatment failure and implant loss, which comes at an additional cost and with additional time and effort for the patient [32].

Management: Whether or not a purulent discharge is present, as well as the degree of soft tissue dehiscence, will determine how to manage premature exposure [33]. An exposure smaller than 3 mm without any purulent exudation does not cause any signs or symptoms in a patient and thus is an occasional finding during postsurgical follow-up. If the exposure happens after the fourth month, the device can be maintained in place with a focused hygiene regimen consisting of topical application of 0.2% chlorhexidine gel twice a day to reduce plaque formation and avoid inflammation of the surrounding tissues [34]. The membrane or mesh needs to be removed right away in cases where the exposure is greater than 3 mm to prevent infection of the regenerating tissue. The flaps should be closed to allow the grafted area to recover for at least 4-5 months if the underlying bone graft is not damaged. To protect the regenerating tissue, the underlying soft tissue must not be lost during removal. Additionally advised is the use of amoxicillin and clavulanic acid for antibiotic coverage [35]. The membrane or mesh must be removed right away once the exposure is accompanied by a purulent discharge to prevent the infection from spreading to the underlying regenerating tissue and causing damage. Then, the graft must be delicately removed to get rid of any infected debris and inflammatory tissue that can compromise the healing process. The recommended dosage for Augmentin (GlaxoSmithKline) is 875 mg of amoxicillin and 125 mg of clavulanic acid twice daily for at least five days [36].

Points to remember: There are some points that are important to remember while dealing with complications of guided bone regeneration. Give the soft tissue enough time to heal before doing a GBR operation. Prior to surgery, all sources of infection (e.g., periodontally, endodontically, or hopelessly involved teeth) must be eliminated [37]. Apply appropriate pre- and postoperative treatment, including systemic and topical antibiotics. Ensure adequate knowledge regarding oral anatomy and the prevention and treatment of complications [38].

Implant Removal

Dental implant removal is rarely necessary. However, when required, it is usually due to severe peri-implantitis, loose dental implants, nerve damage, sinus problems, and loose crowns [39].

**Figure 1:**
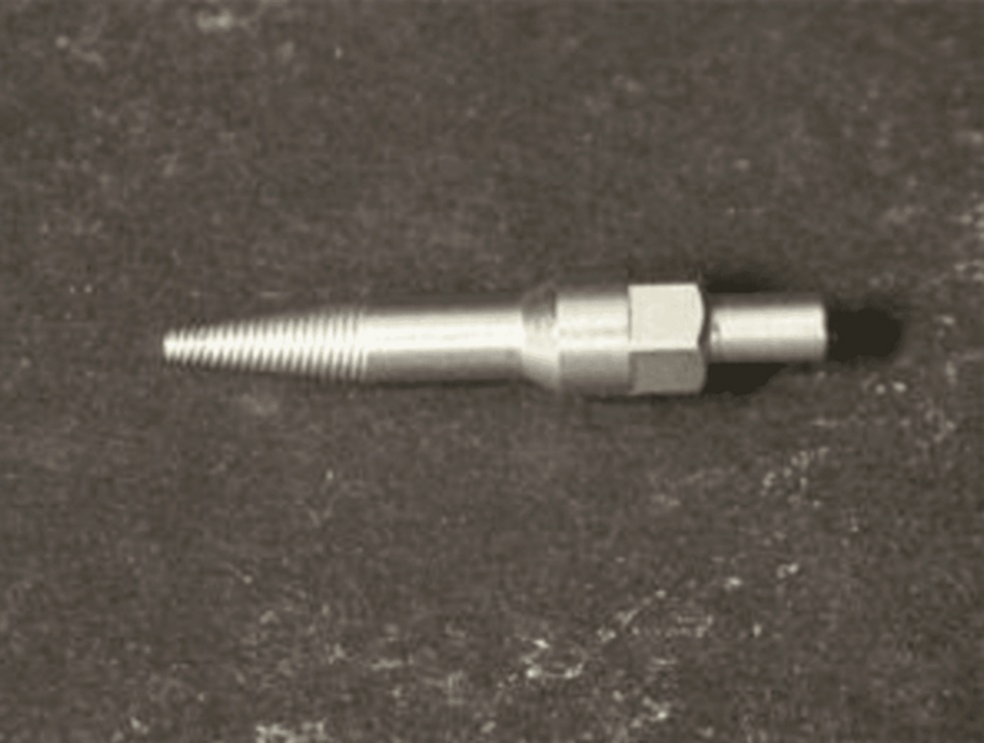
Reverse screw implant retrieval tool Source: [42]

Methods of implant removal: A moveable implant can be easily removed using forceps, the counter-torque ratchet technique (CTRT), or by rotating the implant counterclockwise. The counter-torque ratchet technique (CTRT) and the reverse screw technique (RST) may be helpful where damage to the surrounding tissues is to be avoided. Both of these procedures engage the implant and reverse screw it out of the bone with a counterclockwise force. Little luxation can rotate the surrounding bone and soft tissue with minimal damage and trauma [40]. The use of elevators, forceps, counter-torque ratchets, screw removal tools, piezo tips, high-speed burs, and trephine burs are a few techniques for removing immobile implants. The least intrusive method for removing an implant without harming neighboring structures is the CTRT. The application of CTRT should only be taken into consideration if the implant can be engaged and reverse-torqued until mobile [41]. When a fractured implant’s connection is compromised or when the ratchet cannot be engaged to use the CTRT, the reverse screw technique (RST) should be used to remove the implant [42]. To engage the implant, a screw removal device is employed (Figure 1). The retrieval tool will engage the internal thread and extract the implants using reverse hand torque.

Piezo tips and high-speed burs can be used in conditions where CTRT and RST are not useful to loosen the abutment [43].

Systemic causes

**Figure 2:**
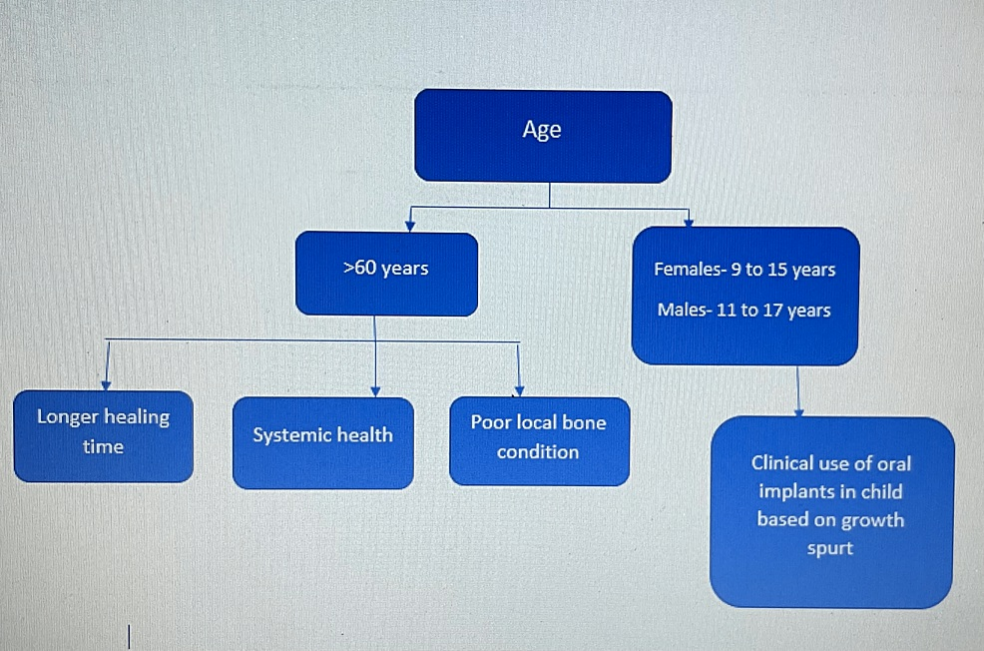
Effect of age on implant health Source: [44]

One of the key factors in implant failure is age. Older individuals have worse local bone problems and longer possible healing durations and are more susceptible to changing systemic health conditions. The chance of an implant failing rises with increasing age (Figure 2) [44].

In smokers, the survival rate of dental implants is reduced. Smoking slows down blood flow because of increased peripheral resistance and platelet aggregation, which has an impact on the osseointegration process. Patients with bruxism experience implant failure more frequently than individuals without parafunction (41% versus 12%). The increased failure risk among bruxers is caused by the implant’s uncontrolled functional loading, which causes micromotions over the critical limit and prevents osseointegration by encasing the implant in fibrous tissue [44]. There is not much proof that diabetes is a direct risk factor for dental implant problems or failure, despite the disease’s high incidence worldwide. Numerous systemic consequences, such as microvascular and macrovascular disorders, impaired wound healing, and increased susceptibility to infection, are linked to diabetes [45]. These circumstances could raise the chance of postoperative problems after dental implant installation. Additionally, a significant risk factor for periodontal disease is diabetes [46]. If diabetes has an adverse effect on osseointegration, it is more likely to harm implants positioned in the maxilla, which has a predominance of cancellous bone, than in the anterior jaw, which has an abundance of cortical bone.

The results of dental implants could be significantly impacted by any alteration in the patient’s overall health or medical condition. Cardiovascular disorders can be the risk factor for implant failure. Through a number of methods, cardiovascular disorders generally have a direct impact on blood flow to tissues. This manifestation alone hinders the healing process and interferes with the delivery of oxygen through blood flow [47]. With enough oxygen present, fibroblast activity, collagen production, capillary expansion, and macrophage activity all increase, helping to keep wounds from becoming infected. This impairs blood circulation and lowers oxygen and nutrition levels. As a result, we can anticipate seeing a possible impact on how the reaction to osseointegration turns out [48].

Osteoporosis causes bones to become weak and brittle, and reduced mineral density (mass/volume unit) in typically mineralized bone is a skeletal condition that characterizes it [49]. The worry that osteoporosis poses a risk factor for dental implants is based on the theory that the mandible and maxilla share the same impaired bone metabolism as other bones in the body [50]. The notion that implant osseointegration may be impacted by osteoporosis-related decreased bone metabolism is also concerning [51].

Certain medications can be one of the factors in implant failure. Corticosteroids are frequently used to treat a variety of systemic illnesses. Their frequent use causes a patient’s immune system to be suppressed, which increases their risk of contracting bacterial, viral, and fungal infections. Patients who use exogenous steroids run the risk of developing osteopenia and osteoporosis, and conventional therapies for these infections might be difficult to administer. This should be kept in mind by the clinician when looking at the maxilla and mandible. Patients on systemic corticosteroid therapy are more likely to experience decreased bone density, increased epithelial fragility, and immunological suppression, all of which affect the dental implant’s ability to osseointegrate. In such situations, it is important to monitor the adrenal gland suppression rate and seek medical attention [52].

Bisphosphonates are a well-known class of medications that act as bone resorption inhibitors by inhibiting osteoclast activity. The osteoclast-mediated bone resorption caused by tumors, which causes hypercalcemia and osteolytic metastases, has been inhibited by a class of intravenous bisphosphonates that contain nitrogen and include pamidronate (Aredia) and zoledronate (Zometa). It was discovered by oral and maxillofacial surgeons that the use of these two intravenous bisphosphonate medications was associated with cases of avascular necrosis (osteonecrosis) of the mandible and/or maxilla.

Management of Systemic Causes of Implant Failure

Diabetes: A complete understanding of the patient’s medical history, present course of treatment, and degree of glycemic control throughout time, as well as limiting surgical therapy in poorly managed diabetic patients, are two fundamental aspects of surgical management for any patient with diabetes. In the last 10-15 years, there has been a significant change in how diabetes mellitus is treated medically [53]. Patients with less-than-optimal glucose control may have a higher risk of developing postoperative complications such as infection or slow wound healing. To avoid such bad occurrences, the dental clinician must get a history from the patient, including earlier HbA1c levels, to determine how well or poorly the patient is controlled [54]. Those diabetic individuals with the worst glycemic control are likely at the most risk for postoperative surgical complications. It is ideal to achieve appropriate glycemic control before implant surgery.

Myocardial infarction: Nitrate premedication, oxygen administration, achievement of profound local anesthesia, stress reduction measures, preoperative pain medication, and patient monitoring of blood pressure and heart rate are preventive measures [55]. Additionally, preserving the patient’s comfort and relaxation may be helped by the use of conscious sedation. Additionally, the dental care provider must be aware of any anticoagulant or thrombolytic treatments being used and comprehend that getting oral implants does not always warrant stopping these treatments [56].

Osteoporosis: Prior to implant surgery, a current medical history should be collected. Patients at risk for metabolic bone disease need to be carefully checked and have their nutrition looked at, and any systemic problems need to be taken care of first [57]. Physiologic calcium (1,500 mg/day) and vitamin D (400-800 IU/day) dosages are advised throughout the postoperative period. Since smoking is a significant risk factor for osteoporosis and implant failure, patients should try to quit smoking and follow a balanced preoperative and postoperative diet [58]. Implant sites should be supplemented before or during implant surgery when there is insufficient bone volume. To avoid overloading the implant and implant loss, the occlusal load should also be evenly distributed across the dentition [59].

Corticosteroids: Despite the lack of evidence to the contrary, patients who get corticosteroid therapy may not be a suitable risk category for implants. First and first, seek medical counsel. Although the validity of the evidence supporting the use of steroids may be questioned, medicolegal and other factors point to the need to err on the side of caution and administer steroids until one is certain that collapse is highly unlikely.

Bisphosphonates: Before starting intravenous bisphosphonate medication, a patient should undergo a complete oral examination and achieve dental stability. Any infection that is still alive must be removed. Prior to administering intravenous bisphosphonates, healing must be complete if any issue necessitates oral surgery, including the placement of dental implants [60]. Patients who are receiving intravenous bisphosphonates for asymptomatic conditions should practice adequate oral hygiene and dental care to avoid dental conditions that might necessitate dentoalveolar surgery. Direct osseous damage procedures are to be avoided. Dental implant placement should be avoided in oncology patients who have received numerous doses of the more strong intravenous treatment (4-12 times per year) [61]. Surgery is not prohibited while oral bisphosphonates are taken, but the dental professional must proceed with caution, and the patient must be made aware of any potential side effects.

## Conclusions

The use of implants is widespread and likely to increase over the next years, which suggests that dental professionals will deal with implant failure and associated consequences more frequently. One must identify the cause to treat the current condition and gain knowledge for future therapies. Timely intervention is always possible with routine checkups. Minimizing the number and severity of issues that will unavoidably arise requires knowledge, learning, and experience. Unfortunately, it is all too true that the final examination frequently comes first, followed by the lesson, which is the problem with utilizing experience as a guide.
